# Supplementary material for: Rapid cortical mapping with cross-participant encoding models
Source: Imaging Neurosci (Camb). 2026 Sep 3;4:IMAG.a.1349. doi: 10.1162/IMAG.a.1349 (PMC13543446; doi:10.1162/IMAG.a.1349)
Supplement: Supplementary file 1 [file IMAG.a.1349_supp.pdf]

Supplemental Material

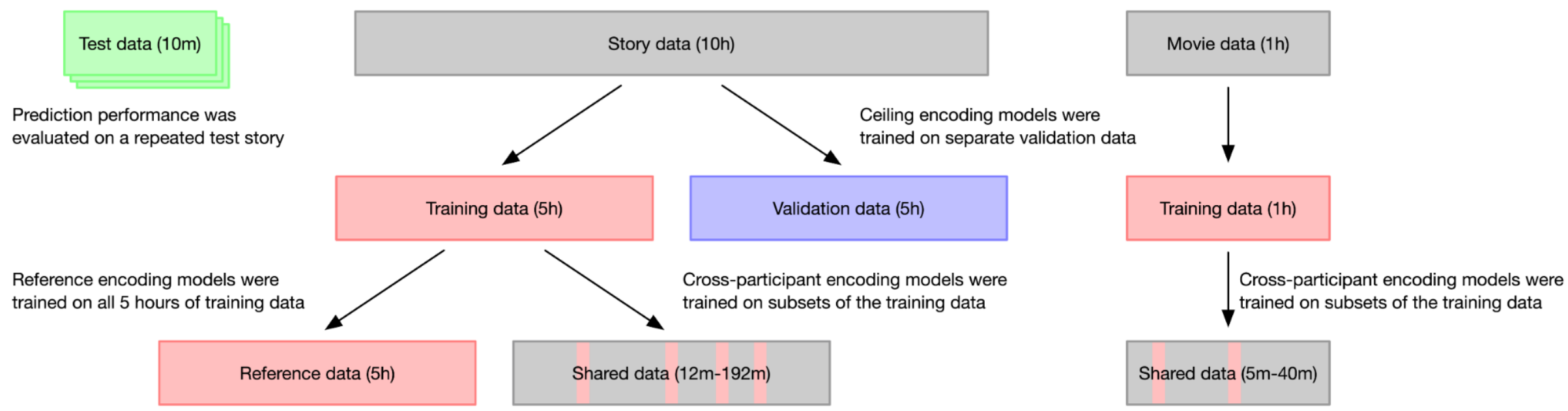

Figure S1. MRI data usage. The 10 hours of story data were partitioned into 5 hours of training data and 5 hours of validation data. The training data were used to train reference encoding models and cross-participant encoding models, while the validation data were used to train ceiling encoding models. All of the training data were used to train reference encoding models, while subsets of the training data were used to train cross-participant encoding models.

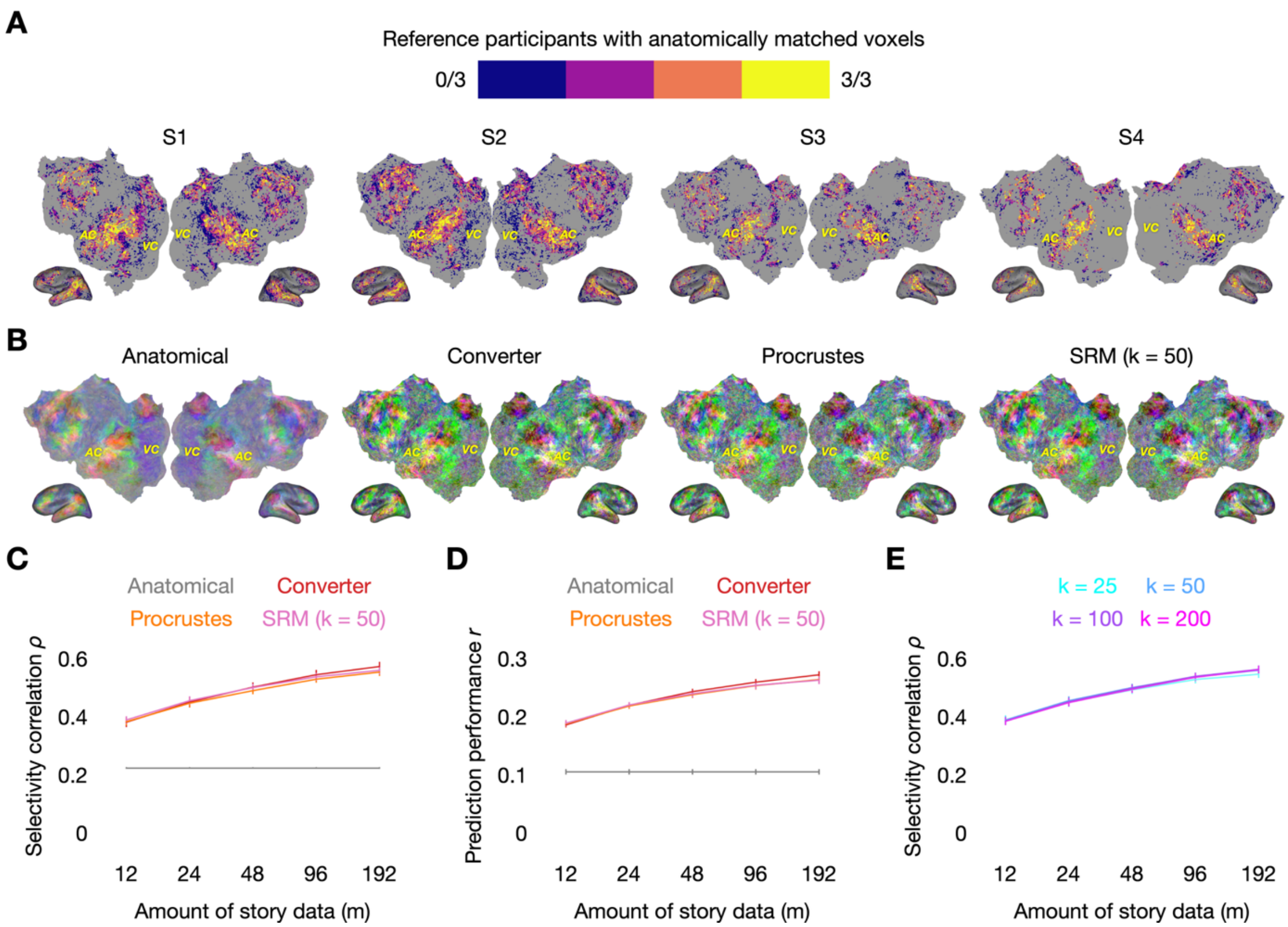

Figure S2. Sensitivity to alignment algorithms. Semantic encoding models were transferred from reference participants to goal participants using anatomical and functional alignment algorithms. (**A**) To assess the anatomical overlap between goal and reference participant voxels, the predictor voxels from the reference participants were anatomically aligned with the predicted voxels from the goal participants. Most predicted voxels from the goal participants had some anatomical correspondence to the predictor voxels from the reference participants. (**B**) Semantic selectivity was visualized by projecting encoding model weights onto the first 3 principal components of a group space. Cross-participant encoding models trained using different functional alignment algorithms (linear converters, pairwise Procrustes hyperalignment, shared response models) captured similar patterns of semantic selectivity. (**C**) Semantic selectivity was operationalized by simulating responses to 10,470 words and ranking the words based on the simulated responses. Cross-participant models using functional alignment had high selectivity correlation with the ceiling model. Cross-participant models using anatomical alignment had much lower selectivity correlation with the ceiling model. (**D**) Prediction performance was quantified using the linear correlation between predicted and actual response time-courses for a held-out test story. Cross-participant models using functional alignment had high prediction performance. Cross-participant models using anatomical alignment had much lower prediction performance. Data points indicate the mean across significantly predicted voxels under the ceiling model. Error bars indicate the standard error of the mean (n = 4 goal participants). (**E**) Shared responses models were trained using different numbers of latent features. Cross-participant encoding models trained using different numbers of latent features captured similar patterns of semantic selectivity.

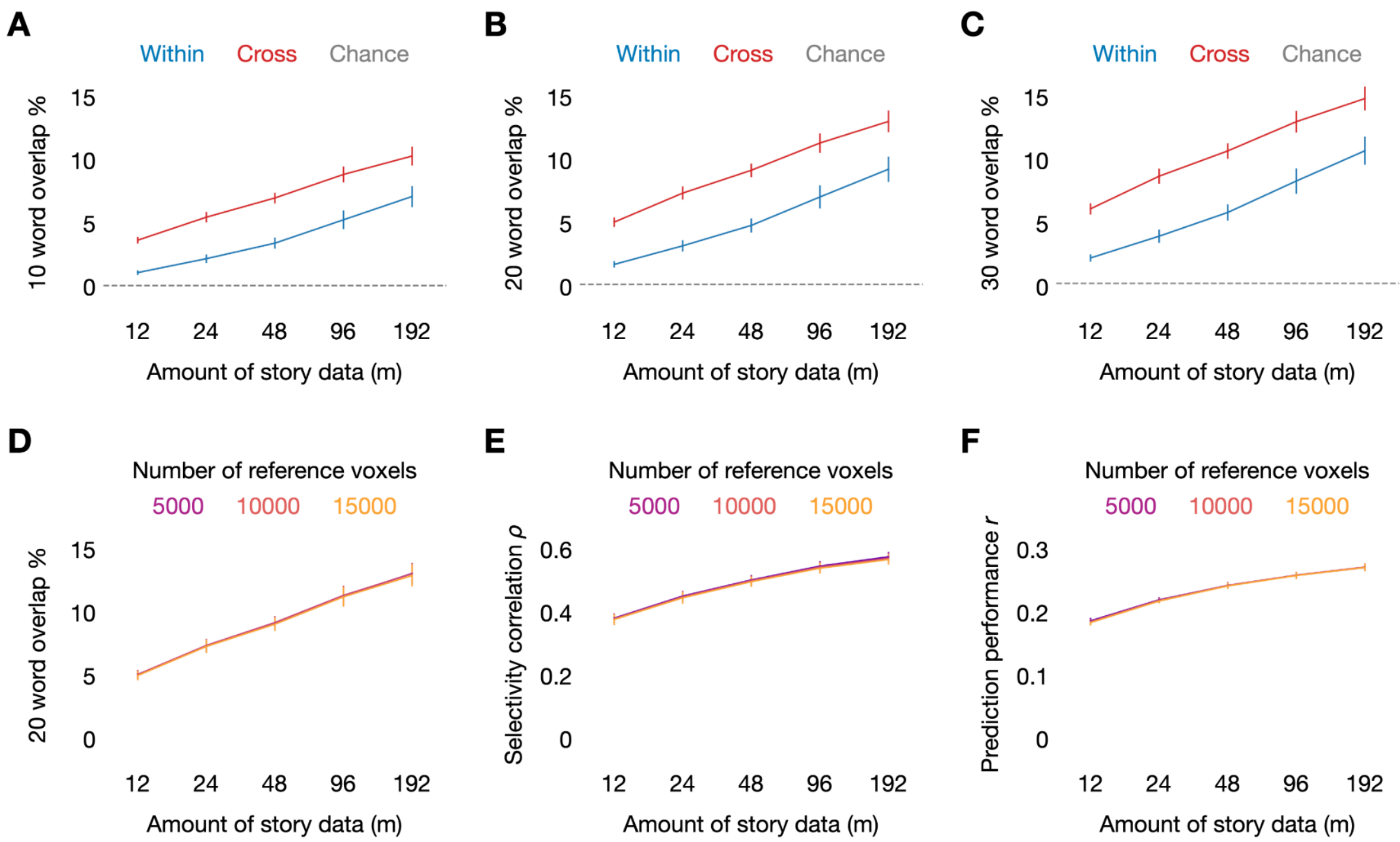

Figure S3. Sensitivity to thresholds. Cross-participant and within-participant semantic encoding models were trained on brain responses to narrative stories. (**A**-**C**) Semantic selectivity was operationalized by simulating responses to 10,470 words and identifying the words with the highest simulated responses. Cross-participant models had higher word overlap with the ceiling model than within-participant models regardless of whether word overlap was computed using the top 10, 20, or 30 words. (**D**) Cross-participant models had higher word overlap with the ceiling model than within-participant models regardless of whether reference encoding models were trained using the top 5,000, 10,000, or 15,000 voxels from each participant. (**E**) Semantic selectivity was alternatively operationalized by simulating responses to 10,470 words and ranking the words based on the simulated responses. Cross-participant models had higher selectivity correlation with the ceiling model than within-participant models regardless of whether reference encoding models were trained using the top 5,000, 10,000, or 15,000 voxels from each participant. (**F**) Prediction performance was quantified using the linear correlation between predicted and actual response time-courses for a held-out test story. Cross-participant models had higher prediction performance than within-participant models regardless of whether reference encoding models were trained using the top 5,000, 10,000, or 15,000 voxels from each participant. Data points indicate the mean across significantly predicted voxels under the ceiling model. Error bars indicate the standard error of the mean (n = 4 goal participants).

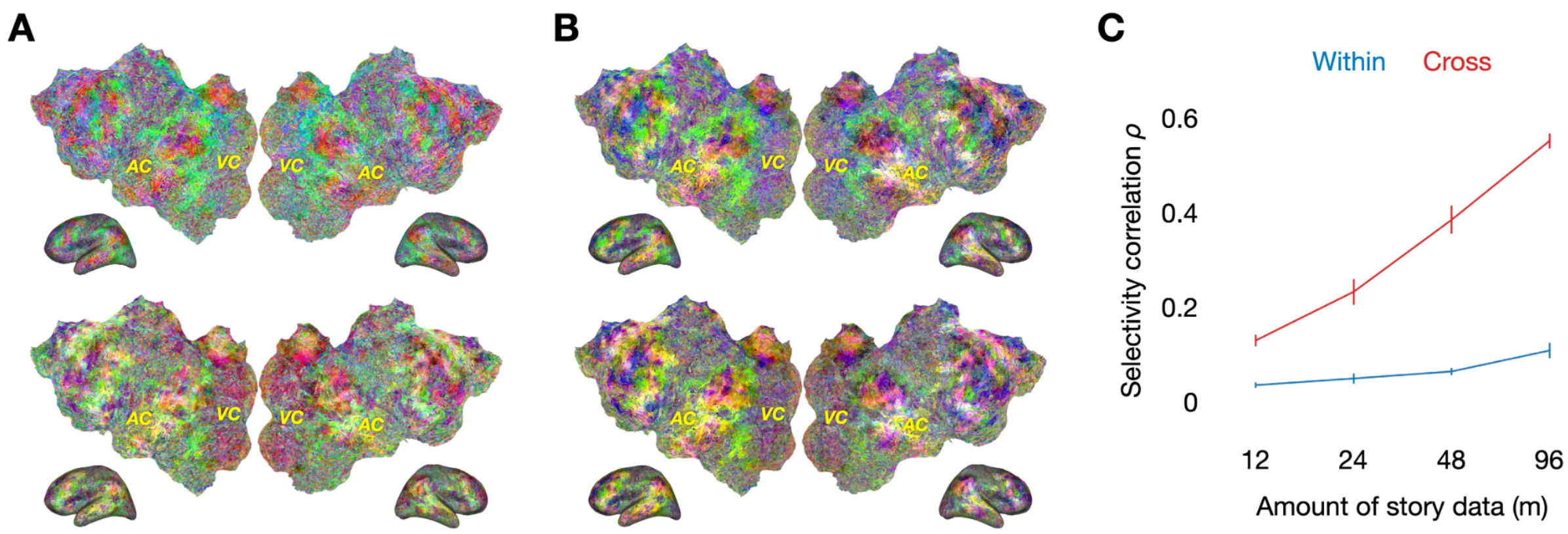

Figure S4. Sensitivity to stimuli. Cross-participant and within-participant semantic encoding models were trained on brain responses to narrative stories. (**A**) Semantic selectivity was visualized by projecting encoding model weights onto the first 3 principal components of a group space. The top and bottom maps were trained on different 24 minute subsets of the story data. Within-participant models produced very different maps when trained on different subsets of the story data. (**B**) Cross-participant models produced more similar maps when trained on different subsets of the story data. (**C**) Encoding models trained on different subsets of the story data were compared to assess reliability. Semantic selectivity was operationalized by simulating responses to 10,470 words and ranking the words based on the simulated responses. Cross-participant models trained on different subsets had higher selectivity correlation than within-participant models. Data points indicate the mean across significantly predicted voxels under the ceiling model. Error bars indicate the standard error of the mean (n = 4 goal participants).

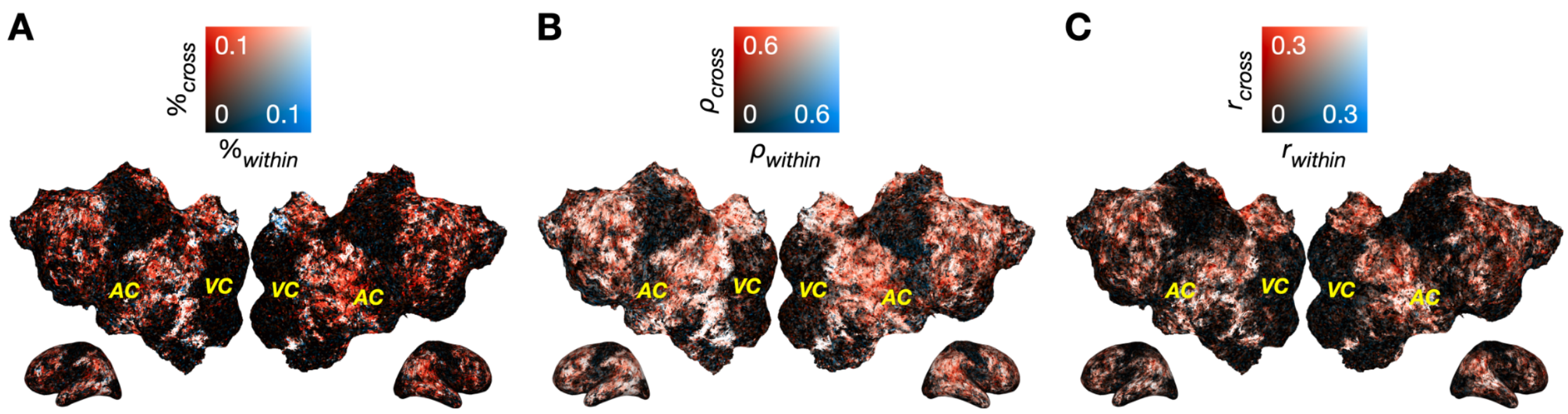

Figure S5. Cross-participant and within-participant modeling across cortex. Cross-participant and within-participant semantic encoding models were trained on brain responses to 24 minutes of narrative stories. (**A**) Semantic selectivity was operationalized by simulating responses to 10,470 words and identifying the 20 words with the highest simulated responses. Cross-participant models had higher word overlap with the ceiling model than within-participant models. (**B**) Semantic selectivity was alternatively operationalized by simulating responses to 10,470 words and ranking the words based on the simulated responses. Cross-participant models had higher selectivity correlation with the ceiling model than within-participant models. (**C**) Prediction performance was quantified using the linear correlation between predicted and actual response time-courses for a held-out test story. Cross-participant models had higher prediction performance than within-participant models.

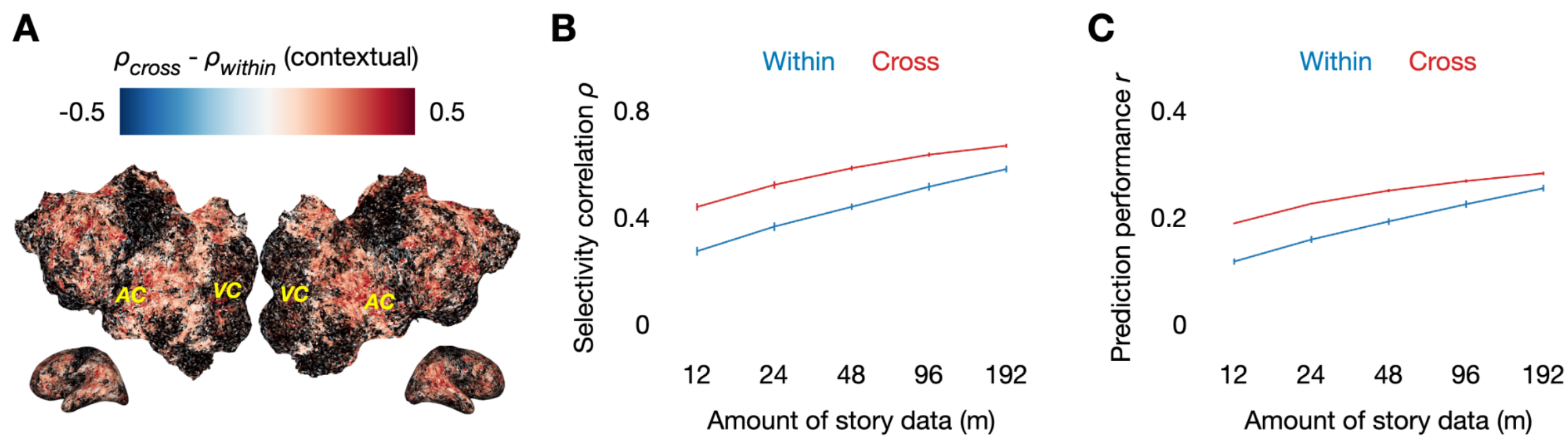

Figure S6. Rapid contextual semantic mapping. Cross-participant and within-participant encoding models were trained on brain responses to narrative stories using contextual semantic features extracted from a GPT language model. (**A**) Semantic selectivity was operationalized by simulating responses to 3,838 segments from an external set of narrative stories and ranking the segments based on the simulated responses. Cross-participant models trained on 24 minutes of data from the goal participant had higher selectivity correlation with the ceiling model than within-participant models trained on 24 minutes of data from the goal participant. (**B**) The advantage of cross-participant modeling decreased as the models were trained on more data from the goal participant. (**C**) Cross-participant models had higher prediction performance than within-participant models trained on the same amount of data from the goal participant, but the advantage decreased as the models were trained on more data from the goal participant. Data points indicate the mean across significantly predicted voxels under the ceiling model. Error bars indicate the standard error of the mean (n = 4 goal participants).

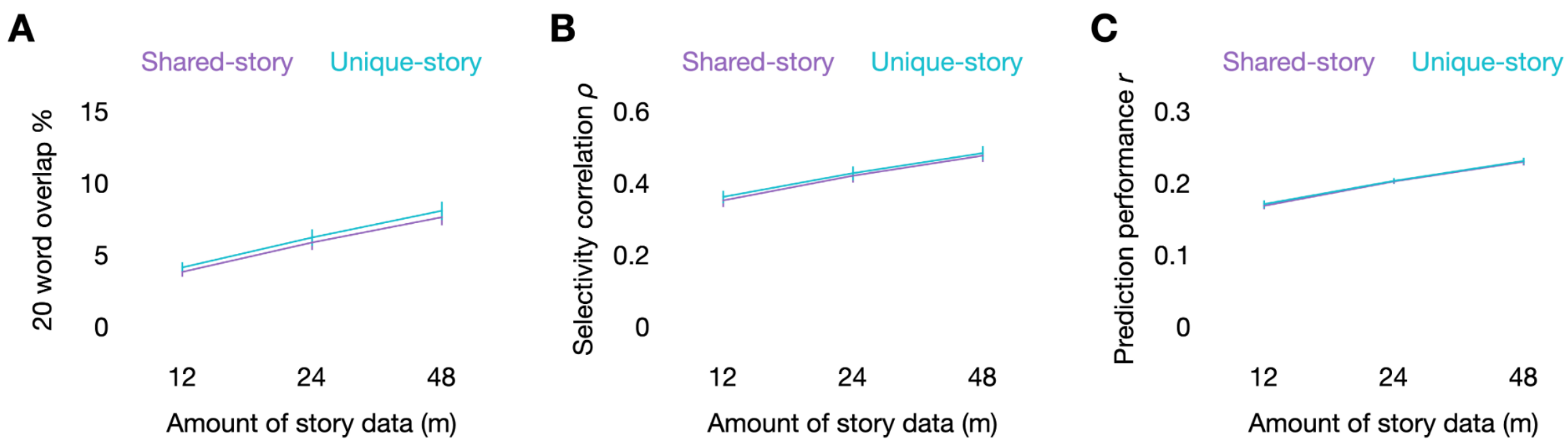

Figure S7. Shared and unique reference stories. Semantic encoding models were trained on 60 minutes of additional story data from each reference participant. The shared-story models used the same 60 minutes of story data for each participant, while the unique-story models used a different 60 minutes of story data for each participant. The unique-story models slightly outperformed the shared-story models, which suggests that varying the stimuli could improve cross-participant modeling. Data points indicate the mean across significantly predicted voxels under the ceiling model. Error bars indicate the standard error of the mean (n = 4 goal participants).

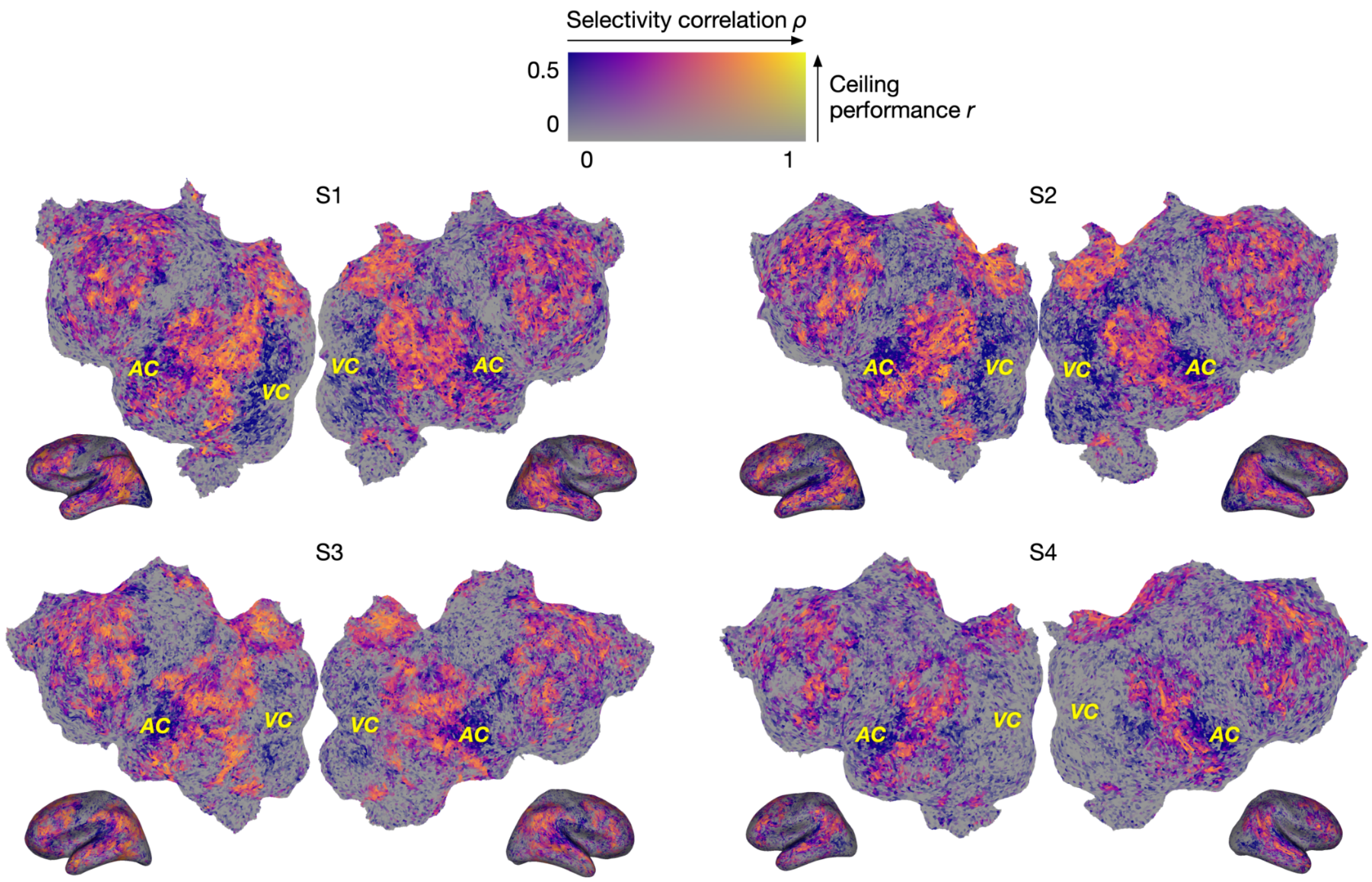

Figure S8. Selectivity correlation of movie-aligned encoding models. Cross-participant semantic encoding models were trained on brain responses to 20 minutes of silent movies. Ceiling encoding models were trained on brain responses to 5 hours of stories. Semantic selectivity was operationalized by simulating responses to 10,470 words and ranking the words based on the simulated responses. Cross-participant models had high selectivity correlation with the ceiling models in most well-predicted regions outside of visual cortex (VC) and auditory cortex (AC).
